# Effect of supplemental dietary phytogenic blends on growth performance, jejunal histomorphometry, and jejunal immunity of broiler chickens

**DOI:** 10.5194/aab-68-13-2025

**Published:** 2025-01-06

**Authors:** Ahmet Ceylan, Pınar Saçaklı, Özge Özgenç Çınar, Muhammad Shazaib Ramay, Umair Ahsan, Josoa André Harijaona, Alev Gürol Bayraktaroğlu, Fabrice Manghebati, Ali Calik

**Affiliations:** 1 Department of Histology and Embryology, Faculty of Veterinary Medicine, Ankara University, Ankara, 06110, Türkiye; 2 Department of Animal Nutrition and Nutritional Diseases, Faculty of Veterinary Medicine, Ankara University, Ankara, 06110, Türkiye; 3 Department of Plant and Animal Production, Burdur Vocational School of Food, Agriculture and Livestock, Burdur Mehmet Akif Ersoy University, Burdur, 15030, Türkiye; 4 Center for Agriculture, Livestock and Food Research, Burdur Mehmet Akif Ersoy University, Burdur, 15030, Türkiye; 5 Manghebati S. A., Zone de la Basse Haye, Châteaubourg, 35220, France

## Abstract

This study evaluated the effects of two phytogenic blends on broiler performance, intestinal histomorphology, CD4^+^ (cluster of differentiation) and CD8^+^ T-cell numbers, and mRNA abundances of several cytokines in broilers. For this purpose, a total of 300 Ross 308 male broiler chicks that were 1 d old were randomly allocated to five experimental groups. The control group was fed a basal diet without any additives, and there were two phytogenic supplement groups (blend A, mainly comprising extracts of *Thymus* *vulgaris* and *Filipendula* *ulmaria*, and blend B, consisting of *Ginkgo* *biloba* and *Silybum* *marianum*) with two dosage regimens each (100 and 200 mg kg^−1^ (denoted A_100_ and A_200_) and 100 and 300 mg kg^−1^ (B_100_ and B_300_) of the diet, respectively). Over the total growing period, body weight gain and feed intake were unchanged among the groups, although phytogenic blend B showed a dose-dependent improvement in feed conversion ratio. Both phytogenic blends did not affect carcass characteristics. Jejunal morphology (villus height, crypt depth, and their ratio) was modified depending on both the composition and the dosage levels of the selected phytogenics. Also, both phytogenic blends linearly increased the CD4^+^ and CD8^+^ T-cell numbers in the jejunum. Moreover, no major treatment effects were observed on mRNA abundances of cytokines (IL-1 β , IL-6, and TNF α ). However, across the two phytogenic additives employed, a positive linear dose response in IL-1 β abundance was noted on day 21 in broilers fed phytogenic blend B. Overall, dietary phytogenic blend B improved the intestinal health and growth performance of chickens compared to blend A. Further studies are suggested to elucidate the effects of the tested phytogenic blends on gut microbiome and on oxidative stress in broiler chickens.

## 1 Introduction

Therapeutic antibiotics have been historically employed to treat infectious diseases in animals and humans alike. However, sub-therapeutic levels of in-feed antibiotics have remained in use as growth-promoting agents in poultry diets to improve productivity. Inclusion of antibiotics in poultry diets as growth promoters was considered an essential component until the realization of antimicrobial resistance in bacteria that reduced the effectiveness of antibiotics. Consequently, this practice of using antibiotics as growth-promoting factors in animal diets attracted a complete ban that led to the development of several alternatives to spare the use of antibiotics (Ahsan et al., 2016). Among these alternatives, independent nutrients, probiotics, prebiotics, organic acids, and additives from botanical sources have been extensively studied. Although studies have reported the replacement potential of antibiotic growth promoters (AGPs) with these alternatives, botanical sources continue to be promising alternatives due to their additional advantages. Plant-based dietary interventions, referred to as phytogenics, usually consist of whole or parts of herbs, spices, or plants in addition to aqueous or alcoholic extracts, essential oils, and/or oleoresins encompassing the bioactive molecules (phytochemicals) of the plant source (Hashemi and Davoodi, 2011; Wang et al., 2024).

Each herb possesses unique phytochemicals, such as polyphenols (quercetin, curcumin, resveratrol), terpenes (eugenol, carvacrol, thymol, capsaicin), or aldehydes (cinnamaldehyde, vanillin), which exert distinct effects on animals. In poultry, dietary supplementation with these phytochemicals (either in pure form or as a herbal blend) has shown various benefits, including redox balance maintenance, intestinal function improvement, immunomodulation, suppression of pathogenic microorganisms, and overall health promotion, thus boosting growth performance (Rossi et al., 2020; Skoufos et al., 2020). The key feature attributed to these phytochemicals is their role in regulating the microbial ecology of the gut by minimizing the pathogenic load, which prevents the loss of intestinal microarchitecture, relieves the burden on the immune system, and allows animals to be at their optimal performance (Hashemi and Davoodi, 2011; Rossi et al., 2020).

The vast variety of plants on the face of the earth has resulted in the development of numerous phytogenics; therefore, the number of studies in this domain has greatly increased over the past years. Moreover, the inclusion levels of phytogenics in poultry are mostly recommended at lower dosages compared to the effective dose measured by in vitro studies (Hafeez et al., 2016), which ensures the safe use in poultry diets. Consequently, the number of phytogenic feed additives has seen an upward trend in the market. Simultaneously, it has contributed to complications in the evaluation of phytogenics. Newman and Cragg (2020) reported that single-source extracts or purified bioactive molecules are less effective for in vitro studies, and these results were further confirmed by in vivo studies. Therefore, a recent trend of blending and mixing phytogenics from different botanical sources has further heightened this discrepancy, since the bioactive components of different sources in the blend may interact in an additive, synergistic, or antagonistic manner despite recent studies reporting synergistic effects among the bioactive molecules (Oso et al., 2019; Pirgozliev et al., 2019). Consequently, conflicting and inconclusive effects of phytogenics have been reported for poultry, which requires optimization in the selection, usage regimens, and application levels of phytogenic blends and mixtures (Hafeez et al., 2016; Ahsan et al., 2018, 2022). Therefore, the efficacy of single and multiple phytogenics remains unresolved in the poultry industry, under both normal and challenging conditions.

Previous studies have demonstrated an improvement in growth performance of broiler chickens in response to the supplemental extracts from different herbal plants, such as ginkgo (Zhang et al., 2012), oregano (Franciosini et al., 2016), thyme (Hashemipour et al., 2013), sage (Rasouli et al., 2020), capsicum (Liu et al., 2021), basil (Thuekeaw et al., 2022), and milk thistle (Bendowski et al., 2022). Evidence also suggests that phytochemicals, present in various herbs and spices, possess digestion-stimulating properties, particularly through the modulation of digestive enzymes (Liu et al., 2021; Rossi et al., 2020). Furthermore, these phytochemicals, especially polyphenolic molecules in herbs, also play a crucial role in strengthening the host's defense system (Pandey et al., 2019). Keeping in view the valuable functional activities of extracts of individual phytochemicals and conflicting reports of beneficial effects of blends of phytogenics in poultry, we investigated the role of two different blended phytogenics as growth promoters in broiler chickens. These blends primarily consisted of *Thymus vulgaris* and *Filipendula ulmaria* extracts (blend A) or *Ginkgo biloba* and *Silybum marianum* (blend B) extracts. Blend A was primarily characterized by thymol, carvacrol, quercetin, kaempferol, epicatechin, tannins, flavone glycosides, flavonoids, and salicylates. Blend B was mainly characterized by silymarin (silybin), quercetin, kaempferol, ginkgolides, bilobides, catechins, flavonolignans, flavone glycosides, terpene lactones, and proanthocyanidins. To the best of our understanding, no study has described the possible effects of these blends on different traits of broiler chickens. Therefore, the objective of our study was to reveal the potential health benefits of these blends at different supplemental levels with special emphasis on intestinal histomorphometry and intestinal immunity using immunohistochemical staining of lymphocytes and mRNA expression of cytokines in the intestine of broiler chickens.

## 2 Materials and methods

### 2.1 Birds and management

This study was approved and conducted under the guidelines of the Institutional Animal Care and Use Committee of Ankara University. A total of 300 Ross 308 male broiler chicks that were 1 d old, obtained from a commercial hatchery, were randomly allocated to five experimental groups, each with six replicate pens, each pen comprising 10 chickens. The group serving as control was fed a basal diet, while other groups received basal diets fortified with 100 or 200 mg kg^−1^ of blend A (A_100_ and A_200_, respectively) or 100 or 300 mg kg^−1^ of blend B (B_100_ and B_300_, respectively). Blend A consisted of *Thymus vulgaris* and *Filipendula ulmaria* extracts primarily characterized by thymol, carvacrol, quercetin, kaempferol, epicatechin, tannins, flavone glycosides, flavonoids, and salicylates, whereas blend B contained *Ginkgo biloba* and *Silybum marianum* extracts mainly characterized by silymarin (silybin), quercetin, kaempferol, ginkgolides, bilobides, catechins, flavonolignans, flavone glycosides, terpene lactones, and proanthocyanidins. Both phytogenic products were supplied by Mangebati (Châteaubourg, France). Basal diets for starter (days 0–14), grower (days 14–28), and finisher (days 28–42) phases of broiler chickens based on corn–soybean meal were formulated to meet or exceed Aviagen (2019) nutrient recommendations (Table 1). Supplemented diets were offered from day 0 of the study.

**Table 1:** Ingredient and nutritional composition of basal diets (%, as fed basis).

Item	Starter	Grower	Finisher
Corn	52.00	54.80	59.12
Soybean meal (47 % CP)	40.00	36.54	31.80
Vegetable oil	3.60	4.70	5.50
Dicalcium phosphate	0.72	0.65	0.63
Limestone	2.34	2.12	1.83
DL-Methionine (98 %)	0.355	0.30	0.275
L-Lysine sulfate (55 %)	0.225	0.17	0.145
L-Threonine	0.11	0.07	0.05
Sodium bicarbonate	0.20	0.20	0.20
Sodium chloride	0.25	0.25	0.25
Vitamin premix^1^	0.10	0.10	0.10
Mineral premix^2^	0.10	0.10	0.10
Nutritional composition (calculated)			
Dry matter	88.91	88.99	89.05
Crude protein	23.03	21.51	19.56
AME_*n*_, kcal kg^−1^	3000	3104	3204
Lysine	1.440	1.275	1.159
Digestible lysine	1.280	1.156	1.022
Methionine + cysteine	1.065	0.971	0.894
Digestible methionine + cysteine	0.963	0.874	0.804
Threonine	0.988	0.893	0.797
Digestible threonine	0.858	0.770	0.685
Calcium	0.960	0.871	0.781
Available phosphorus	0.480	0.441	0.392

^1^ Provided per kilogram of complete diet: vitamin A, 12 000 IU (international unit); vitamin D_3_, 2500 IU; vitamin E, 40 IU; vitamin K_3_, 5 mg; thiamin, 2.5 mg; riboflavin, 6 mg; pyridoxine, 5 mg; pantothenic acid, 15 mg; niacin, 25 mg; folic acid, 1 mg; biotin, 50 µ g; vitamin B_12_, 20 µ g. ^2^ Provided per kilogram of complete diet: Cu, 5 mg; I, 1 mg, Co, 200 µ g; Se, 150 µ g; Fe, 60 mg; Zn, 60 mg; Mn, 80 mg. Note that CP represents crude protein, and AME_*n*_ represents apparent metabolizable energy (nitrogen-corrected).

Chickens were housed in a controlled environment throughout the study. An automatic ventilation system was used to maintain the relative humidity between 50 % and 60 %. Temperature was maintained at 33 °C for the first 3 d and then gradually reduced by approximately 3 °C each week until it reached 22 °C, which was maintained thereafter. The lighting schedule was 24 h of light and 0 h of dark (24L : 0D) for the first 3 d, 23L : 1D from day 4 to day 7, and 20L : 4D from day 8 until the end of the study. A plastic feeder and a nipple drinker line were installed in each pen to provide ad libitum access to feed and water. Fresh wood shavings were used as litter.

All chickens were individually weighed, and feed intake (FI) was recorded at weekly intervals. Any mortality was recorded (including bird weight) on a daily basis to adjust for the evaluation of growth performance. Body weight gain (BWG), FI, and feed conversion ratio (FCR) were subsequently calculated to evaluate the growth performance of broiler chickens.

### 2.2 Sampling procedures

On day 21 and day 42, one bird from each replicate was selected based on average pen body weight for analyses; it was euthanized by cervical dislocation, and the intestinal tract was immediately removed. Tissue samples, 1 cm in length, were obtained from the jejunum's middle section for histomorphometric and immunohistochemical analyses. An additional sample was collected from the jejunum and flash-frozen (snap-frozen) in liquid nitrogen to assess the mRNA abundances of several cytokines. Subsequently, the weights of carcass, carcass parts, and organs were recorded on day 42. Carcass yield was calculated relative to the slaughter weight, whereas organ and carcass part yields were calculated relative to the carcass weight.

### 2.3 Jejunal histomorphometry

Jejunum samples were fixed in 10 % neutral-buffered formalin, dehydrated in a series increasing concentrations of ethanol, cleared with xylol, and finally embedded in paraffin for microscopic examination. The intestinal segments were cut into 5 µ m thick sections using a microtome (Leica RM2125 Leica Microsystems GmbH, Wetzlar, Germany). To evaluate jejunal morphometry, Masson's trichrome stain as modified by Crossmon was applied to cross-sections, enabling the visualization of cellular structures and accurate determination of tissue morphology (Culling et al., 1985). Jejunal sections were examined under a light microscope (Leica DM2500, Leica Microsystems GmbH, Wetzlar, Germany) coupled with a digital microscope camera (Leica DFC450, Leica Microsystems GmbH, Wetzlar, Germany). Subsequently, the images were generated, and histomorphometric measurements were carried out using the ImageJ software (US National Institutes of Health, Bethesda, MD). For these measurements, a total of 10 well-oriented villi and crypts were randomly selected from each section. Villus height (VH) was measured from the top of the villus to the crypt mouth, and crypt depth (CD) was defined as the depth of the invagination between adjacent crypt mouths.

### 2.4 Immunohistochemistry for CD4^+^ and CD8^+^

Jejunum samples were fixed in 10 % neutral-buffered formalin for 18 h, embedded in paraffin, and sliced into 4 µ m sections using a microtome for subsequent immunohistochemistry (IHC) analysis. IHC staining was performed using the standard streptavidin–biotin complex method. Serial sections were processed concurrently. After the deparaffinization and rehydration steps, endogenous peroxidase activity was blocked by using 3 % hydrogen peroxide solution for 15 min. To remove the methylene bridges between proteins, a heat-induced epitope retrieval method was employed using sodium citrate solution (10 mM, pH 6). Sequentially, sections were incubated with 10 % normal rabbit serum for 30 min for protein blocking, followed by incubation with anti-CD4 (1 : 400, Bioss Technology Co. Ltd.) and anti-CD8 α (1 : 500, Bioss Technology Co. Ltd.) primary antibody for 2 h at 37 °C. After a TBS (Tris-buffered saline) wash, sections were incubated with biotinylated anti-rabbit antibody (IgG BA1000, Vector Laboratories Inc., CA, USA) for 30 min. Following another washing step, a peroxidase-conjugated streptavidin reagent (Standard Vectastain Elite ABC Kit, PK-6100, Vector Laboratories Inc., CA, USA) was added for 30 min, and then sections were incubated in a peroxidase substrate solution DAB (3,3^′^-diaminobenzidine substrate, SK-4100, Vector Laboratories Inc., CA, USA). The sections were then counterstained with Gill's (III) hematoxylin and a coverslip applied. Images of the specimens were captured using a Leica DM2500 light microscope equipped with a DFC450 digital camera. Image analysis was performed using ImageJ software (US National Institutes of Health, Bethesda, MD). Two independent observers (blind to sample identities) randomly selected and analyzed 10 non-overlapping fields of view per tissue section. The staining intensity threshold was set to distinguish positively stained cells from the background. Inter-observer reliability was assessed using the intraclass correlation coefficient. Discrepancies in counts were resolved through joint re-evaluation.

### 2.5 Total RNA extraction and reverse transcription

A 50 mg aliquot of jejunal tissues was weighed into a 2 mL microcentrifuge tube and homogenized in 900 µ L TRI reagent (Zymo Research, Irvine, CA, USA) using a FastPrep-24™ (MP Biomedicals, Santa Ana, CA, USA). Total RNA was extracted from the homogenate using the Direct-zol RNA Miniprep Plus Kit (Zymo Research, Irvine, CA, USA), following the manufacturer's recommendations. The total RNA concentration was determined at an optical density (OD) of 260 (NanoDrop 2000, Thermo Fisher Scientific, Waltham, MA, USA), and RNA purity was verified by evaluating the 260/280 OD ratios. After extraction, 2 µ g of total RNA was used to synthesize first-strand cDNA using the OneScript Plus cDNA Synthesis Kit (Applied Biological Materials Inc., Richmond, BC, Canada) according to the manufacturer's recommendation, and the cDNA was stored at - 20 °C.

### 2.6 Quantitative real-time PCR

The mRNA abundance of cytokines (interleukin (IL)-1 β , IL-6, and tumor necrosis factor α (TNF α )) was determined by a CFX Connect real-time PCR system (Bio-Rad Laboratories, Inc., CA, USA), using BlasTaq™ 2 × qPCR MasterMix (Applied Biological Materials Inc., Richmond, BC, Canada). Details of primer sets are provided in Table 2. The cDNA was diluted 1 : 5 in nuclease-free water, and 4 µ L of the diluted cDNA was added to each well of a 96-well plate. Subsequently, 16 µ L of real-time PCR master mix, comprising 10 µ L of BlasTaq™ 2 × qPCR MasterMix, 1 µ L each of 10 µ M forward and reverse primers, and 4 µ L of sterile nuclease-free water per reaction, was added to each well for a final volume of 20 µ L. During the PCR reaction, samples underwent an initial enzyme activation at 95 °C for 3 min, followed by 40 cycles of denaturation at 95 °C for 15 s and annealing and extension at 60 °C for 1 min. Product specificity was confirmed by analyzing the melting curves. The mRNA abundance was analyzed using glyceraldehyde 3-phosphate dehydrogenase (GAPDH) as an endogenous control. Average mRNA abundance relative to GAPDH for each sample was calculated using the 2^−ΔΔCt^ method (Livak and Schmittgen, 2001). The calibrator for each gene was the average Δ Ct value from the control group for each sampling day.

**Table 2:** Sequences of primer pairs used for the amplification of target and reference genes.^1^

Gene^2^	Primer sequence	Size (bp)	Acc (reference)
IL-1 β	CCCGCCTTCCGCTACA	66	NM_204524.1
	CACGAAGCACTTCTGGTTGATG		
IL-6	GCTTCGACGAGGAGAAATGC	63	NM_204628.2
	GGTAGGTCTGAAAGGCGAACAG		
TNF α	CCCATCCCTGGTCCGTAAC	77	XM_040694846.2
	ATACGAAGTAAAGGCCGTCCC		
GAPDH	CCTAGGATACACAGAGGACCAGGTT	64	NM_204305
	GGTGGAGGAATGGCTGTCA		

^1^ For each gene, the primer sequence for forward (F) and reverse (R) (5^′^–3^′^) primers, the amplicon size (bp), and the NCBI (National Center for Biotechnology Information) accession number (Acc) used for the primer design are listed. ^2^ IL: interleukin, TNF α : tumor necrosis factor α , GAPDH: glyceraldehyde 3-phosphate dehydrogenase.

### 2.7 Statistical analysis

The data were analyzed using the ANOVA procedure of SPSS software, version 14.01 (SPSS Inc., Chicago, IL, USA). One-way ANOVA was employed to assess the effects of phytogenic blend supplementation, and significant means were separated using Tukey's test. Additionally, polynomial contrasts were applied to evaluate both linear and quadratic effects. Statistical differences were considered significant at P≤0.05 , and results were presented as mean ± pooled standard error of the mean.

## 3 Results

### 3.1 Growth performance and carcass characteristics

Growth performance of broiler chickens remained largely unaffected among the groups (Table 3) except FCR at day 0–42, which was improved in the B_300_ group compared to the control ( P=0.019 ). Besides these, a linear improvement in FCR at day 28–42 ( P=0.006 ) and day 0–42 ( P=0.001 ) was seen in broiler chickens fed increasing supplemental levels of phytogenic blend B. No significant mortality was noted among the treatments throughout the study. Carcass, carcass part, and edible giblet yields were not different among the groups (Table 4).

**Table 3:** Growth performance of broiler chickens fed different levels of different phytogenic blends.^1^

Item^3^	Growth performance traits^2^											
	BWG, g				FI, g				FCR			
	days 0–14	days 14–28	days 28–42	days 0–42	days 0–14	days 14–28	days 28–42	days 0–42	days 0–14	days 14–28	days 28–42	days 0–42
Control	453.0	1242.6	1767.9	3463.4	577.42	1619.9	2940.5	5137.8	1.278	1.305	1.663	1.485^a^
A_100_	462.6	1324.1	1759.9	3546.6	589.01	1708.3	2898.8	5196.0	1.272	1.293	1.647	1.468^ab^
A_200_	455.4	1272.5	1745.7	3473.5	574.16	1681.3	2823.4	5078.8	1.262	1.320	1.618	1.463^ab^
B_100_	451.7	1281.3	1726.3	3459.2	576.23	1679.9	2799.9	5056.2	1.277	1.312	1.623	1.460^ab^
B_300_	447.0	1280.6	1764.5	3492.1	573.94	1655.4	2815.4	5044.8	1.283	1.293	1.598	1.447^b^
SEM	2.66	9.96	14.61	17.57	4.36	11.16	21.71	27.11	0.009	0.006	0.008	0.004
P value	0.476	0.134	0.910	0.535	0.825	0.122	0.175	0.375	0.957	0.601	0.088	0.019
Polynomial contrasts												
Control vs. herbal blend A												
P linear	0.792	0.348	0.660	0.858	0.809	0.083	0.092	0.461	0.566	0.493	0.088	0.061
P quadratic	0.294	0.025	0.943	0.125	0.267	0.062	0.769	0.213	0.947	0.316	0.788	0.539
Control vs. herbal blend B												
P linear	0.565	0.171	0.756	0.722	0.821	0.147	0.057	0.289	0.913	0.700	0.006	0.001
P quadratic	0.697	0.709	0.408	0.640	0.911	0.252	0.472	0.888	0.851	0.366	0.641	0.683

^a, b^ Means with different superscripts in the same column are significantly different ( P<0.05 ). ^1^ Data represent the mean value of six replicate pens of 10 birds per pen. ^2^ Abbreviations: BWG, body weight gain; FI, feed intake; FCR, feed conversion ratio. ^3^ Control: corn–soybean meal basal diet; A_100_ and A_200_: *Thymus vulgaris* and *Filipendula* *ulmaria* extract supplemental groups at levels of 100 and 200 mg kg^−1^ of the diet, respectively; B_100_ and B_300_: *Ginkgo biloba* and *Silybum marianum* extract supplemental groups at levels of 100 and 300 mg kg^−1^ of the diet, respectively. Note that SEM represents the standard error of the mean.

**Table 4:** Carcass and carcass part yields (%) of broiler chickens fed different levels of different phytogenic blends.^1^

Item^2^	Carcass	Thigh	Breast	Wings	Liver	Heart	Spleen	Gizzard	Bursa of fabricius	Abdominal fat
Control	74.09	21.60	24.68	7.23	1.80	0.49	0.10	1.19	0.20	0.78
A_100_	74.89	21.74	24.35	6.94	1.77	0.46	0.11	1.10	0.19	0.89
A_200_	75.56	21.43	25.39	6.90	1.68	0.43	0.10	1.06	0.21	0.92
B_100_	74.51	22.28	24.20	7.28	1.83	0.43	0.12	1.16	0.19	0.83
B_300_	74.90	21.58	24.40	7.01	1.76	0.48	0.12	1.16	0.18	0.96
SEM	0.25	0.18	0.25	0.06	0.03	0.01	0.004	0.03	0.007	0.05
P value	0.452	0.612	0.598	0.190	0.543	0.246	0.160	0.510	0.862	0.775
Polynomial contrasts										
Control vs. herbal blend A										
P linear	0.075	0.756	0.432	0.122	0.262	0.096	0.711	0.167	0.783	0.283
P quadratic	0.927	0.637	0.386	0.500	0.769	0.978	0.187	0.742	0.635	0.709
Control vs. herbal blend B										
P linear	0.315	0.730	0.551	0.442	0.779	0.414	0.120	0.658	0.468	0.372
P quadratic	0.790	0.203	0.628	0.254	0.456	0.115	0.274	0.913	0.833	0.591

^1^ Data represent the mean values of six replicates per treatment. ^2^ Control: corn–soybean meal basal diet; A_100_ and A_200_: *Thymus vulgaris* and *Filipendula* *ulmaria* extract supplemental groups at levels of 100 and 200 mg kg^−1^ of the diet, respectively; B_100_ and B_300_: *Ginkgo biloba* and *Silybum marianum* extract supplemental groups at levels of 100 and 300 mg kg^−1^ of the diet, respectively.

### 3.2 Jejunal histomorphometry

**Figure 1:**
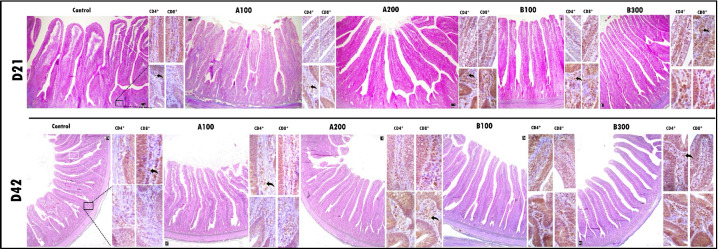
The larger images depict the histomorphometric characteristics of a cross-section of the jejunum using trichrome staining. Side images are immunohistochemical examinations of lamina propria of the villus and crypt. The immunohistochemical examination revealed the presence and distribution of CD4^+^ and CD8^+^ T cells within the lamina propria of both the villus and crypt regions among the groups. The scale bars in the images represent 50 µ m in the larger images and 20 µ m in the side images.

Histomorphometry of broiler chickens is presented in Table 5 and Fig. 1. At day 21 and day 42, the B_300_ group had greater VH in comparison with the control ( P=0.001 and P=0.041 , respectively). Supplemental phytogenic blends B_100_ and B_300_ increased the CD of broiler chickens at day 21 compared to the control ( P=0.003 ). A dose-dependent linear increase was seen in VH and CD at day 21 in response to supplemental phytogenic blends A ( P=0.003 and P=0.033 , respectively) and B ( P=0.001 and P<0.001 , respectively). However, the villus height to crypt depth (V : C) ratio at day 21 was not different among the groups. On day 42, increasing levels of dietary phytogenic blend B linearly improved the jejunal VH of broiler chickens ( P=0.003 ). Broiler chickens in the B_300_ group had greater CD at day 42 than those in other groups except the control ( P≤0.001 ). This increase was seen quadratically with increasing supplemental phytogenic blend B ( P≤0.001 ). The V : C ratio at day 42 was greater in the A_100_ group than that of the control group ( P=0.015 ). However, increasing linear and quadratic responses were noted in V : C at day 42 with increasing supplemental levels of phytogenic blends A and B compared to the control ( P<0.05 ).

**Table 5:** Jejunal histomorphometry of broiler chickens fed different levels of different phytogenic blends.^1^

Item^3^	day 21			day 42		
	Villus height, µ m	Crypt depth, µ m	V : C ratio^2^	Villus height, µ m	Crypt depth, µ m	V : C ratio^2^
Control	799.1^b^	134.0^b^	6.07	1261.7^b^	188.2^ab^	6.80^b^
A_100_	971.9^ab^	160.7^ab^	6.18	1532.2^ab^	166.1^b^	9.61^a^
A_200_	973.2^ab^	158.7^ab^	6.24	1475.7^ab^	165.8^b^	9.14^ab^
B_100_	956.0^ab^	176.2^a^	5.53	1395.2^ab^	155.4^b^	9.14^ab^
B_300_	1106.5^a^	173.6^a^	6.53	1556.1^a^	212.4^a^	7.41^ab^
SEM	23.38	4.09	0.15	35.08	5.09	0.33
P value	0.001	0.003	0.359	0.041	<0.001	0.015
Polynomial contrasts						
Control vs. herbal blend A						
P linear	0.003	0.033	0.676	0.067	0.075	0.040
P quadratic	0.063	0.167	0.936	0.102	0.300	0.089
Control vs. herbal blend B						
P linear	0.001	<0.001	0.710	0.003	0.391	0.030
P quadratic	0.252	0.189	0.084	0.229	<0.001	0.001

^a, b^ Means with different superscripts in the same column are significantly different ( P<0.05 ). ^1^ Data represent the mean values of six replicates per treatment. ^2^ Abbreviation: V : C ratio, villus height to crypt depth ratio. ^3^ Control: corn–soybean meal basal diet; A_100_ and A_200_: *Thymus vulgaris* and *Filipendula ulmaria* extract supplemental groups at levels of 100 and 200 mg kg^−1^ of the diet, respectively; B_100_ and B_300_: *Ginkgo biloba* and *Silybum marianum* extract supplemental groups at levels of 100 and 300 mg kg^−1^ of the diet, respectively.

### 3.3 Jejunal CD4^+^ and CD8^+^ T-cell population

At day 21, the B_300_ group had more CD4^+^ cells in the jejunum compared to other groups except the B_100_ group ( P<0.001 ; Table 6 and Fig. 1). The control group had a lower jejunal CD4^+^ population compared to other groups ( P<0.001 ). In addition, the jejunal CD4^+^ population was greater in the B_100_ group than the A_100_ group ( P<0.001 ). The jejunal population of CD8^+^ cells at day 21 was lower in the control group compared to other groups ( P<0.001 ), whereas the B_100_ and B_300_ groups had a higher CD8^+^ cell population than other groups ( P<0.001 ). Besides these, the A_200_ group had more CD8^+^ cells than the A_100_ and control groups ( P<0.001 ). Increasing supplemental levels of phytogenic blends A and B linearly increased the jejunal CD4^+^ and CD8^+^ cell populations compared to the control group ( P<0.001 ). However, the ratio of CD4^+^ cells to CD8^+^ cells was not different among the groups.

**Table 6:** Jejunal CD4^+^ and CD8^+^ T-cell abundances of different levels of different phytogenic blends.^1^

Item^2^	day 21			day 42		
	CD4^+^	CD8^+^	CD4^+^/CD8^+^	CD4^+^	CD8^+^	CD4^+^/CD8^+^
Control	24.10^d^	30.30^d^	0.80	29.43^c^	33.43^d^	0.88
A_100_	28.33^c^	32.87^c^	0.86	32.23^c^	38.40^c^	0.84
A_200_	30.47^bc^	35.57^b^	0.86	37.13^b^	41.67^bc^	0.89
B_100_	33.03^ab^	41.40^a^	0.80	39.27^ab^	44.47^ab^	0.88
B_300_	36.90^a^	42.37^a^	0.87	41.03^a^	47.27^a^	0.87
SEM	0.89	0.90	0.014	0.87	0.96	0.011
P value	< 0.001	< 0.001	0.241	< 0.001	< 0.001	0.689
Polynomial contrasts						
Control vs. herbal blend A						
P linear	< 0.001	< 0.001	0.211	< 0.001	< 0.001	0.780
P quadratic	0.404	0.915	0.379	0.162	0.331	0.158
Control vs. herbal blend B						
P linear	< 0.001	< 0.001	0.075	< 0.001	< 0.001	0.838
P quadratic	0.491	0.009	0.076	0.331	0.548	0.769

^a–d^ Means with different superscripts in the same column are significantly different ( P<0.05 ). ^1^ Data represent the mean values of six replicates per treatment. ^2^ Control: corn–soybean meal basal diet; A_100_ and A_200_: *Thymus vulgaris* and *Filipendula ulmaria* extract supplemental groups at levels of 100 and 200 mg/kg of the diet, respectively; B_100_ and B_300_: *Ginkgo biloba* and *Silybum marianum* extract supplemental groups at levels of 100 and 300 mg kg^−1^ of the diet, respectively.

At day 42, jejunal CD4^+^ cells were lower in the control and A_100_ groups compared to other groups ( P<0.001 ), whereas the A_200_ group had a lower CD4^+^ cell population than the B_300_ group ( P<0.001 ). The control group had a lower CD8^+^ cell population compared to other groups ( P<0.001 ), while the B_300_ group had a higher jejunal CD8^+^ cell population than other groups except the B_100_ group ( P<0.001 ). In addition, the CD8^+^ cell population was greater in the B_100_ group than the A_100_ group ( P<0.001 ). Increasing supplemental levels of phytogenic blends A and B linearly increased the jejunal CD4^+^ and CD8^+^ cell populations compared to the control group ( P<0.001 ). Nonetheless, the CD4^+^ to CD8^+^ cell ratio remained unaffected across the groups.

### 3.4 mRNA abundances of jejunal IL-1 β , IL-6, and TNF α

The mRNA abundances of IL-1 β , IL-6, and TNF α are presented in Table 7. The mRNA abundance of most cytokines was not different among the groups except IL-1 β at day 21. Broiler chickens in the B_300_ group exhibited a significantly greater IL-1 β mRNA abundance ( P=0.004 ) in the jejunum compared to the control and A_100_ groups on day 21. A linear dose-dependent increase in mRNA abundance of IL-1 β at day 21 was seen in broiler chickens fed phytogenic blend B compared to the control ( P=0.005 ).

**Table 7:** Jejunal mRNA abundances of IL-1 β , IL-6, and TNF α of broiler chickens fed different levels of different phytogenic blends.^1^

Item^2^	day 21			day 42		
	IL-1 β	IL-6	TNF α	IL-1 β	IL-6	TNF α
Control	1.07^b^	1.11	1.04	1.07	1.13	1.08
A_100_	1.10^b^	1.15	0.97	0.98	1.16	0.81
A_200_	1.48^ab^	1.70	1.00	0.86	1.33	1.18
B_100_	1.95^ab^	1.79	1.72	0.85	1.14	1.17
B_300_	2.42^a^	1.77	1.80	0.87	1.14	1.12
SEM	0.14	0.13	0.14	0.08	0.10	0.11
P value	0.004	0.277	0.139	0.911	0.968	0.847
Polynomial contrasts						
Control vs. herbal blend A						
P linear	0.125	0.178	0.893	0.522	0.567	0.814
P quadratic	0.431	0.491	0.816	0.969	0.814	0.382
Control vs. herbal blend B						
P linear	0.005	0.079	0.117	0.262	0.970	0.821
P quadratic	0.741	0.609	0.814	0.692	0.984	0.832

^a, b^ Means with different superscripts in the same column are significantly different ( P<0.05 ). ^1^ Data represent the mean values of six replicates per treatment. ^2^ Control: non-supplemented diet; A_100_ and A_200_: *Thymus vulgaris* and *Filipendula ulmaria* extract supplemental groups at levels of 100 and 200 mg kg^−1^ of the diet, respectively; B_100_ and B_300_: *Ginkgo biloba* and *Silybum marianum* extract supplemental groups at levels of 100 and 300 mg kg^−1^ of the diet, respectively.

## 4 Discussion

Modern commercial broilers are routinely exposed to a myriad of environmental stressors and pathogens that negatively impact their performance and overall health. Although AGPs have been used for decades to overcome such challenges, increasing concerns over antimicrobial resistance have prompted a search for alternative strategies, such as the incorporation of phytogenics in poultry diets. Dietary phytogenic additives are usually supplemented at lower inclusion levels than their effective dose computed from in vitro studies (Hafeez et al., 2016), ensuring the safety of bioactive molecules in poultry. Despite the promising results reported in previous studies regarding the use of dietary phytogenics, either alone or in combination, there are still conflicting and inconclusive findings. These discrepancies are likely due to possible additive interactions, synergism, or antagonism between phytogenics, as well as a lack of effective usage level (Vaou et al., 2022). However, most phytogenic additives and bioactive molecules have non-interactive, additive, and synergistic interactions (Vaou et al., 2022). In this context, the present study aimed to evaluate the effect of two well-balanced phytogenic blends at different supplemental levels on growth performance and intestinal health of broiler chickens.

In our study, the B_300_ group had better growth performance in terms of overall FCR. While there was a linear improvement in FCR of broiler chickens in B_100_ and B_300_, blend A had no effect compared to the control. These differences could be related to phytogenic dose used in diets or their composition. Similar to our observations, earlier studies have described conflicting reports that include the growth enhancement effects of different dietary phytogenics in broiler chickens (Gheisar et al., 2015; Wati et al., 2015; Gheisar and Kim, 2018; Hassan et al., 2018; Movahhedkhah et al., 2019; Basit et al., 2020a, b), whereas several other studies reported that different dietary phytogenics were unable to improve the growth performance of broiler chickens (Hafeez et al., 2016; Ahsan et al., 2018; Zabek et al., 2020; Ahsan et al., 2022). The differences in the findings might be attributable to the differences in diets, composition, concentrations of phytogenics, their bioactive molecule concentrations, rearing conditions, or the subjugation of broiler chickens to physiological or microbiological stressors. In our study, growth performance was not different among the groups in the early growth phases. It might be associated with insufficient digestive enzyme-secreting capacity of the gut, releasing relatively lower amounts of digestive enzymes than later growth phases (Khattak et al., 2014). The improvement in overall FCR of broiler chickens due to supplemental phytogenics seems to have occurred due to the improvement in the nutrient digestion and absorption. This idea is supported by the fact that a numerical decrease in FI was noted in phytogenics supplemented groups especially in the finisher growth phase that was reflected as a strong linear tendency in the phytogenic groups compared to the broiler chickens fed diets without phytogenics, although BWG remained largely unaffected across the groups. The improvement in FCR was possibly seen due to improved digestion and absorption of essential nutrients with increasing supplemental levels of phytogenics. The improvement in the digestion and absorption of nutrients in the supplemented groups might be due to improved gut microarchitecture and immunity as seen in our study. Consistent with this notion, previous studies have reported an improvement in the growth performance of poultry following dietary supplementation of phytogenics consisting of extracts of *Silybum marianum* (Morovat et al., 2016; Shahsavan et al., 2021; Shanmugam et al., 2022; Elnesr et al., 2023), *Thymus vulgaris* (Hashemipour et al., 2013, 2016; Ibrahim et al., 2021), and *Ginkgo biloba* (Cao et al., 2012; Yu et al., 2015; Zhang et al., 2012; Ren et al., 2018) via enhancement in nutrient digestibility and increased activity of digestive enzymes.

Carcass yield and relative organ weights did not show significant differences among the treatments. Previous studies also suggested that phytogenic supplementation had no effect on carcass traits (El-Ashram and Abdelhafez, 2020; Feshanghchi et al., 2022). Unlike these, other studies have reported improvements in carcass yields (Hashemipour et al., 2016; Jahanian et al., 2017; Morovat et al., 2016) and organ weights of broiler chickens fed diets supplemented with phytogenics (Li et al., 2022). Niu et al. (2017) noted a linear increase in the eviscerated yield of broiler chickens fed fermented *Ginkgo biloba* leaves. Additionally, there is evidence of lower slaughter efficiency and less abdominal fat in broilers supplemented with *Silybum marianum* extracts (Bendowski et al., 2022; Morovat et al., 2016). Such varied responses could be attributed to differences in the phytogenic substances used, their form, and their purity. In general, carcass, carcass part, and edible giblet yields are directly associated with the live weight of broiler chickens. Modern broiler chickens have been selected for faster growth, heavier body weight, and greater part yields (especially breast meat). Heavier chickens tend to have heavier parts; however, the yields usually remain largely unaffected unless the restriction of nutrients is applied, particularly for those essential in nature. In the present study, all groups received basal diets with ad libitum access, and BWG was not different among the groups; therefore, carcass yields and characteristics were similar.

The gastrointestinal tract is a complex system, and maintenance of structural and functional homeostasis of the intestine is crucial for effective nutrient absorption, defense against infections, and efficient performance. Significant associations between gut villus structures (VH, CD), immune markers (CD3^+^ T lymphocytes), and broiler performances have been demonstrated at a commercial level (Rysman et al., 2023). In the present study, significant changes in the jejunal morphology of broilers were observed following supplementation with phytogenics. Our findings suggest a positive association between the dosage levels of the additives and the rate of increase in VH and CD, indicating the potential for improved nutrient absorption and intestinal health. On day 21, no dose-dependent increments in V : C ratios were noted. However, on day 42, supplementation of both phytogenics modified the balance between VH and CD, leading to a linear increase in the V : C ratio in relation to dosage. These results are supported by previous studies showing positive effects of various phytogenics on histomorphometric aspects of the jejunum (villus height, V : C ratio), when broilers were fed diets supplemented with *Ginkgo* *biloba *oil or fermented leaves (El-Kasrawy et al., 2023; Zhang et al., 2013), silymarin (Jahanian et al., 2017, 2021), and a thymol and carvacrol mix (Li et al., 2022). Galli et al. (2020) stated that a herbal blend is more effective in modifying intestinal V : C ratio than a single herb. The correlation between animal performance and gut health is universally accepted. Many researchers assert that the deepening of intestinal crypts is usually a product of increased enterocyte turnover (proliferation, migration, apoptosis), facilitating longer villi, resulting in higher nutrient absorption, and potentially providing a more robust defense mechanism against pathogen attacks. However, the continuous demand for new tissues requires energy. Therefore, a moderate enterocyte turnover rate (higher V : C ratio) is desired, involving lower maintenance requirements and leaving more energy for weight gain (Teng et al., 2021; Van Nevel et al., 2005). In the present study, on day 42, phytogenics-induced increments in V : C ratios in the intestine of broilers accompanied by improved FCR in the finishing period once again demonstrate a dynamic interaction between these parameters. Recently, in experiments with broilers fed *Ginkgo biloba*-based additives, researchers (Niu et al., 2019; Ren et al., 2018) indicated amelioration in the utilization of nutrients and energy by animals. Hashemipour et al. (2016) also reported better nutrient retention in broilers fed diets supplemented with a mixture of thymol and carvacrol. Previous studies have also reported *Ginkgo biloba*-induced decrements in apoptosis incidences in the intestinal mucosa (Yu et al., 2015), as well as the presence of higher numbers of functional and mature enterocytes (Zhang et al., 2013). In the present study, a comparative assessment of both tested phytogenics revealed that the *Ginkgo biloba*- and *Silybum marianum*-based formulation is a more potent stimulus for intestinal architecture development in broilers. The exact mechanism by which these phytogenics exerted the improvement in the jejunal microarchitecture is not known. However, the improvement in jejunal histomorphometry of broiler chickens is speculated to have occurred due to the antimicrobial and immunomodulatory effects of the bioactive components of supplemented phytogenic blends that reduced the intestinal microbial load and colonization of pathogenic bacteria, thereby sparing the nutrients for the cellular turnover and the development of enterocytes, adding to the elongation of villi and deeper crypts.

The improvement in structural integrity of intestines is directly linked to the maintenance of gut immunity. Phytogenics, owing to their active components, can enhance the gut immunity through immune cell proliferation and the modulation of inflammation, thus contributing to better gut health and growth performance of broiler chickens. To this end, gut immunity of broiler chickens was taken into consideration in terms of T lymphocytes and cytokines. T helper (CD4^+^) and T cytotoxic (CD8^+^) lymphocytes, crucial elements of the intestinal immunological system, play a vital role in regulating the adaptive immune system (Kallon et al., 2013). The present study indicated that the dietary supplementation of both phytogenics contributed to the modulation of cellular immunity by CD4^+^ and CD8^+^ T-cell infiltration in the jejunum compared to the control group. The existing literature suggests that the heightened presence of CD4^+^ and CD8^+^ T cells may play a role in reducing the intestinal colonization of *Salmonella* (Haghighi et al., 2008; Huang et al., 2013), leading to enhanced protection against immunological challenges. This effect is particularly relevant in young animals with immature intestinal functions, making them more susceptible to pathogen exposure. The present study demonstrates that immune modulation can be effectively achieved by using different combinations of plant extracts from distinct sources, as well as by adjusting the dosage levels of these extracts. Balenović et al. (2024) also reported a favorable immunomodulatory effect of a *Cannabis sativa*-based phytogenic additive on cell-mediated and humoral immune responses in broilers via increased CD4^+^ and CD8^+^ lymphocyte subpopulations. In our study, a linear dose-dependent increase in the number of CD4^+^ and CD8^+^ T cells in the jejunum of broiler chickens fed phytogenics was noted, which was more pronounced in those fed phytogenic blend B than phytogenic blend A. In addition, the number of CD8^+^ lymphocytes was usually greater than the number of CD4^+^ T cells throughout the study. Our findings are in line with previous studies reporting the greater number of CD8^+^ cells than CD4^+^ cells in the spleen (Hanieh et al., 2010) and intestine (Huang et al., 2013; Jiang et al., 2015) of broiler chickens fed different phytogenics. Further in the study, mRNA abundances of pro-inflammatory cytokines (IL-1 β , IL-6, and TNF α ) were evaluated to elucidate the effects of the tested phytogenics on immune functions. Depending on the diversity of active ingredients and their concentrations, phytogenics may support immune function by regulating the expression of pro-inflammatory mediators or modulating the anti-inflammatory milieu in an organism (Miguel, 2010; Rossi et al., 2020). In accordance, birds in the B_100_ and B_300_ groups exhibited a positive linear dose-dependent increase in mRNA abundance of IL-1 β on day 21. IL-1 β is considered a key immunomodulator that promotes innate immunity against invading microorganisms and activates cells of the adaptive immune system that are attracted to the infection sites (Weber et al., 2010). Taken together, it is speculated that broiler chickens fed diets supplemented with phytogenic blend B had an effective cell-mediated adaptive defense and a pro-inflammatory innate immune response at an early age, improving their resistance against diverse classes of pathogens. However, no substantial changes were recorded in the mRNA abundances of IL-6 or TNF α in the jejunum of broiler chickens on either day 21 or day 42 with dietary supplementation of both phytogenics. An earlier study reported that the expression of cytokines (IL-1 β , IL-6, IFN- γ ) increased to higher levels in garlic-extract-fed groups compared to ginger (Elmowalid et al., 2019). *Ginkgo biloba *leaves have proven effective in modulating inflammatory mediators in broiler chickens subjected to stress (Zhang et al., 2013). Moreover, addition of an encapsulated mixture of thymol and carvacrol in broiler diets downregulated the jejunal mRNA expression levels of NF- κ B, IL-1 β , and TNF α , whereas IL-6 and IL-10 levels showed no changes (Li et al., 2022). Ibrahim et al. (2021) reported upregulation of IL-10 and IL-2 and downregulation of IL-6 in splenic tissues of broiler chickens fed a 1 % thymol nano-emulsion. Immunomodulatory effects of phytogenics employed in different studies could be attributed to their capability of modifying intestinal microbiota in favor of the host animal, promoting a particular set of cell-signaling proteins or other chemicals, eventually leading to an improved immune response (Rakoff-Nahoum and Medzhitov, 2008; Vidanarachchi et al., 2013).

## 5 Conclusions

In conclusion, dietary supplementation of broiler diets with phytogenic blends A and B at different inclusion levels represents a promising non-drug alternative. Despite the similarities in most effects observed for both phytogenic additives investigated in the study, the phytogenic blend B, which contains extracts of *Ginkgo biloba* and *Silybum marianum*, exhibited a greater potential for improving the growth performance of broiler chickens in terms of FCR, conceivably due to its positive impact on gut immune functions and histomorphological features. Given the potential synergistic effects of these blends, future research focusing on the gut microbiome and protection against oxidative stress is required to fully reveal the benefits of using these blends.

## Data Availability

The data will be made available by the corresponding author on reasonable request.
